# Sonodynamic therapy and magnetic resonance-guided focused ultrasound: new therapeutic strategy in glioblastoma

**DOI:** 10.1007/s11060-023-04333-3

**Published:** 2023-05-14

**Authors:** Lapo Bonosi, Silvia Marino, Umberto Emanuele Benigno, Sofia Musso, Felice Buscemi, Kevin Giardina, Rosamaria Gerardi, Lara Brunasso, Roberta Costanzo, Domenico Gerardo Iacopino, Rosario Maugeri

**Affiliations:** 1 Department of Biomedicine Neurosciences and Advanced Diagnostics, School of Medicine, Neurosurgical Clinic, AOUP “Paolo Giaccone”, Post Graduate Residency Program in NeurologiSurgery, University of Palermo, 90127 Palermo, Italy; 2 IRCCS Centro Neurolesi Bonino-Pulejo, Messina, Italy

**Keywords:** Foscused Ultrasound, Gliomas, Glioblastoma, Sonodynamic Therapy, MRgFUS

## Abstract

Glioblastoma (GB) is one of the most aggressive and difficult-to-treat brain tumors, with a poor prognosis and limited treatment options. In recent years, sonodynamic therapy (SDT) and magnetic resonance focused ultrasound (MRgFUS) have emerged as promising approaches for the treatment of GB. SDT uses ultrasound waves in combination with a sonosensitizer to selectively damage cancer cells, while MRgFUS delivers high-intensity ultrasound waves to precisely target tumor tissue and disrupt the blood–brain barrier to enhance drug delivery. In this review, we explore the potential of SDT as a novel therapeutic strategy for GB. We discuss the principles of SDT, its mechanisms of action, and the preclinical and clinical studies that have investigated its use in Gliomas. We also highlight the challenges, the limitations, and the future perspectives of SDT. Overall, SDT and MRgFUS hold promise as novel and potentially complementary treatment modalities for GB. Further research is needed to optimize their parameters and determine their safety and efficacy in humans, but their potential for selective and targeted tumor destruction makes them an exciting area of investigation in the field of brain cancer therapy.

## Introduction

Gliomas represent about 25% of all primary brain tumors, encompassing malignant and not malignant subtypes [1]. IDH-wildtype glioblastoma (2022 WHO grade 4) exhibited the highest age-adjusted incidence rates and is considered the most aggressive variant, characterized by an extremely aggressive biological behavior resulting in a poor outcome [2]. Despite the enormous progress in biotechnology and medicine field, life expectancy of glioblastoma (GB) patients has improved only slightly over the last 30 years.

As a matter of fact, if untreated, GB’s median survival means is 3 months [3]. Interestingly, the standard management of GB has not changed since Roger Stupp published his work [4].

Recently, supramarginal resection or, where possible, excision of the hyperintense area in FLAIR sequences in MRI (so-called Flair-ectomy) has been shown to be associated with improvement in both overall survival (OS) and progression free survivor (PFS), although not always executable, especially in lesions involving eloquent brain areas [5, 6].

Other therapeutic strategies tested included anti-angiogenic therapy and immunotherapy, though they did not show significant improvement in OS [7]. Some authors also advocated the importance of palliative care to increase the quality of life in patients affected from this tremendous tumor [8, 9].

In the era of genomic and molecular genetics, in which it is possible to investigate all the potential metabolic landscape of the disease, new treatment strategy is going to rely more on biochemical and immunological treatments [10–12].

Various new treatments have been proposed over the years, regarding use of CAR T cells [13], molecular agents enhancing the effect of radiotherapy (RT) [14], up to the application of high and low intensity focus ultrasound [15].

In this context, sonodynamic therapy (SDT) seems to become a promising treatment, offering the possibility of non-invasively eradicate solid tumor in a site-directed manner, employing compounds that become cytotoxic after being exposed to low intensity ultrasound [16]. The importance of this kind of treatment could gain an added value considering the possibility of deep lesion-targeting thanks to the significant depth that low-intensity ultrasound penetrates tissue, not damaging surround brain parenchyma and the chance of aiming directly to cancer stem cells (found to be vital cells for proliferation, differentiation, and treatment resistance of the GB) [17–20]

Remarkably, the possibility of impairing the brain blood barrier (BBB) is another weapon in neurosurgical armamentarium, making easier the access of chemical agents [20, 21].

Our review aims to provide a current view of the use of focused ultrasound, and particularly SDT, in the management of GB, starting with their mechanism of action *in vitro* and in animal models and ending with current or future clinical trials, exploring the limitations and potential of such treatment.

## Materials & Methods

### Search of the literature

**Fig. 1:**
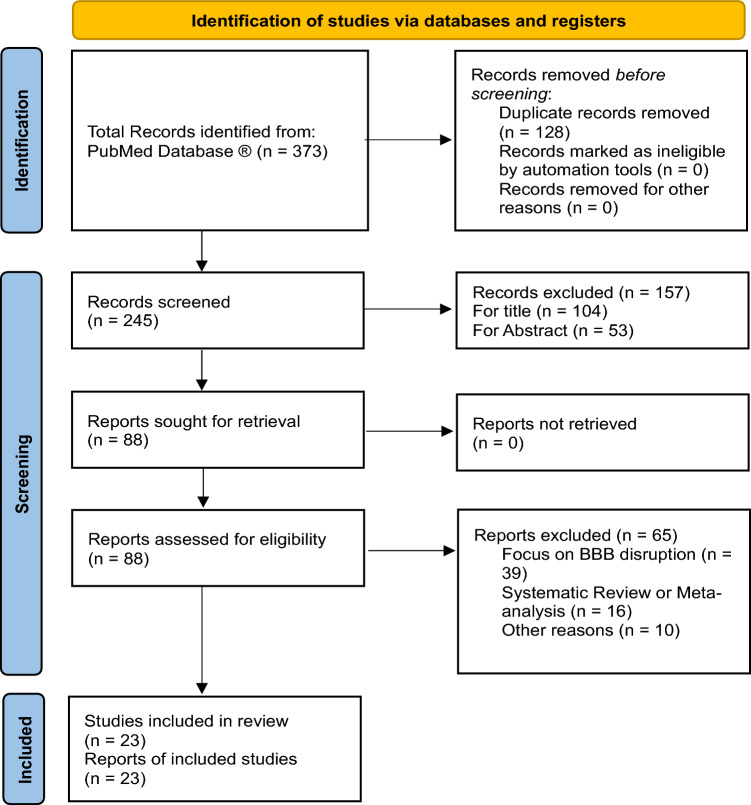
PRISMA Flow Diagram

Preferred Reporting Items for Systematic reviews and Meta-analyses guidelines (PRISMA) were followed to conduct and report this systematic literature review [22] (Fig. 1).

We performed a broad systematic literature search in different online scientific libraries (Pubmed/MEDLINE, Cochrane Library and ClinicalTrial.Gov) for all studies investigating the efficacy and feasibility of SDT in GB treatment. The protocol of this review has been prospectively registered in Open Science Framework and it is publicly available online at https://doi.org/10.17605/OSF.IO/FW4QS.

We searched for studies published up to the 15th of September 2022 without backward limits, using the following MeSH terms “Glioblastoma” AND “Focused Ultrasound”, “Glioblastoma” AND “FUS”, “Glioblastoma” AND “MRgFUS”, “Glioblastoma” AND “Sonodynamic therapy“, “Glioblastoma” AND “High intensity Focused Ultrasound”, “Glioblastoma” AND “HIFU”, “Glioblastoma” AND “Sonodynamic” AND “therapy”, “Gliomas” AND “Sonodynamic therapy“, “Glioblastoma” AND “focused ultrasound” AND “sonosensitizer”, “Glioma” AND “focused ultrasound” AND “sonosensitizer”.

To avoid the potential omission of relevant studies we also manually screened reference lists of articles included and previous systematic reviews and meta-analyses regarding the topic. Duplicate articles were eliminated using Microsoft Excel 16.37 (Redmond, WA, USA).

### Study selection

The studies included in our paper were both studies *in vitro* and *in vivo* using animal models, and ongoing clinical trials. The proof-of-concept of our research was trying to understand the effect of SDT on Glioma/GB cell line and then the feasibility, efficacy, and safety of its application firstly in animal model and then in the clinical practice, evaluating the ongoing clinical trials.

The research strategy initially relied on title and abstract analysis. The article’s full text was retrieved for further investigation if the title and abstract met the inclusion criteria. The data collection process was conducted without using any automated tools. The research was conducted by two different authors separately (U.E.B and K.G) and eventually refined by a third author (L.B). No ethical approval was required for this study.

### Eligibility criteria

The articles were selected according to the following inclusion criteria:

- Full article in English.
- Studies in a preclinical phase (‘in vitro’ and ‘in vivo’ study).
- Case report, case series, retrospective study, prospective study and clinical trials.
- Patients age ≥ 18.
- Patients affected by glioblastoma treated with SDT.

Exclusion criteria:

- Articles not in English.
- Editorials, books, systematic reviews, and meta-analysis.
- Patients age < 18.
- Patients treated with focused ultrasound used to perform a disruption in brain-blood-barrier (BBB).
- Studies evaluating focused ultrasound therapy but not focusing on SDT.

### Data extraction

According to the criteria above, all articles were identified by two reviewers (K.G. and U.E.B.). In case of a discrepancy, a third author (L.B.) arbitrated until a consensus among the authors was reached.

The extracted data included the following: publication’s year, author, study design, patients’ number, patients’ mean age and gender, type of cells or animals, aim of the study and results of the study.

## Results

### Data selection

Our initial research carried out through Pubmed identified a total of 373 articles. We excluded 128 duplicated articles, then we performed a further screening based upon title and abstract reading, eliminating 157 articles.

Finally, after a full text reading and a detailed examination, 65 articles were excluded, because either they were focusing only on the effect of focused ultrasound on BBB disruption (39 articles) or they were reviewing previous scientific works (16 articles) or lastly because they were not inherent with the purpose of this review, including finally 23 studies in our systematic review, according to PRISMA flow diagram inclusion criteria.

The characteristics of included articles are the following ones: publication’s year, author, study design, type of cells and animal model (respectively for the ‘in vitro’ and for the ‘in vivo’ studies), aim of the study and results of the study.

The articles were eventually divided in three tables, including studies ‘in vitro’ and studies ‘in vivo’ on animal models (Tables 1, 2).

**Table 1:** Summary of ‘in vitro’ studies included in the review

References	Study design	Pubblication year	Type of cells	Aim	Parameters	Results	Outcome
Hayashi et al. [23]	*In vitro*	2009	U105MG and U251MG glioma cells	To investigate the efficacy of low-intensity ultrasound on malignant astrocytic tumor cells using Photofrin as a photosensitizer	Intensity: 0.3W/cm^2^	Photofrin-SDT enhanced US cell killing in LRP/α2-MR-expressing glioma cells	higher cell killing was observed following SDT: 52.7 ± 17.5%,13.0 ± 4.6%, and 3.9 ± 0.9% for 5, 15 and 30 s, respectively in comparison to US alone treatment
					Frequency: 0.95 MHz		
					Duration of 5, 15, or 30 s		
Xu et. al. [24]	*In vitro*	2012	U251 glioma stem-like cells	To compare the susceptibility of GSCs and U251 to SDT using Photofrin as a sonosensitizer	Frequency: 1.0 MHz	Cell viability and apoptosis assays showed that SDT damaged both GSCs and U251 glioma cells, but GSCs were significantly less susceptible	in the SDT groups, GSCs had a significantly lower apoptosis rate (p. < 0.01) than the U251 glioma cells (63.43 ± 8.07% vs. 38.85 ± 10.24%)
					Intensity:0.5W/cm^2^		
					Timing: 1 min		
Hao et al. [25]	*In vitro*	2014	C6 glioma cells	To investigate the role of intracellular calcium overload in the *in vitro* apoptosis of C6 glioma cells mediated by low level ultrasound and hematoporphyrin monomethyl ether (HMME) therapy	Frequency: 0.5 MHz	The combined use of low-level ultrasound and HMME improved the apoptotic rate of C6 glioma cells mediated by ultrasound alone; it was showed that Ca2 overload plays a role in the SDT-induced apoptosis	The SDT treatment in calcium-supplemented medium showed significant increase in apoptosis (49.4 ± 2.6%) in comparison to control group (apoptotic rate < 5%)
					Intensity: 1.0 W/cm^2^		
					Timing: 1 min		
Gonzales et al. [26]	*In vitro*	2016	F9 glioma cells	To evaluate the efficacy of FUS activation of the sonosensitizer AIPcS2a(*Aluminum phthalocyanine disulfonate)* together with Bleomycin	Frequency: 1 MHz	FUS activation of the sonosensitizer AlPcS2a together with BLM significantly inhibits the ability of treated glioma cells to grow as three-dimensional tumor spheroids	SDT at an intensity of 0.6 W/cm^2^ causes a reduction in spheroid volume by about 30%. In contrast, FUS alone at the same intensity leads to a reduction in spheroid volume of less then 10%
					Intensity: 0.2, 0.4, or 0.6 W/cm^2^		
					Timing: 3 min		
Chen et al. [27]	*In vitro*	2017	C6 glioma cells	To evaluate the effects of combination of application of temozolomide (TMZ) together with SDT	Frequency:1 MHz	The combination between TMZ together with SDT enhanced the expression of mitochondrial pathway apoptosis proteins, as well as suppressed MMP-2 to weaken the migration ability in TMZ-resistant C6 cell line and by decreasing NHE1 protein expression	The survival rates are 96.8 ± 7.9%, 94.5 ± 6.6%, 61.1 ± 13.3%,30.1 ± 4.4%. 28.5 ± 2.9%,26.3 ± 4.1%, 25.4 ± 4.2%, 19.3 ± 3.8% after 1, 2, 4, 6, 8, 12, 18, 24 h insonication respectively. Cell viability in SDT group was < 60% vs. 100% in control group
					Intensity: 1 W/cm^2^		
					Timing: N/A		
Sun et al. [28]	*In vitro*	2018	U373 human glioma cells and NIH 3T3 murine fibroblastic cells	To evaluate the killing effect of SDT in human glioma cells by using sinoporphyrin sodium used as sonosensitizer	Frequency:1.0 MHz	The combined use of SDT with DVDMS reduced human glioma cells viability and apoptosis; no significant changes were observed in the viability of normal cells	The cell viability was measured as 87.27%, 90.81% and 37.76% in the 2 microM DVDMS alone, 0.45 W/cm^2^ ultrasound and SDTgroup, respectively
					Intensity: 0.45 W/cm^2^		
					Timing: 1 min		
Dai et al. [29]	*In vitro*	2019	C6 glioma cells	To investigate the apoptotic effect and mechanisms of SDT mediated by hematoporphyrin monomethyl ether (HMME) on C6 glioma cells *in vitro*	Frequency:1 MHz	SDT and HMME combination increased the mitochondrial signal pathway apoptosis; moreover, it was proved an increased production of ROS and a reduction of MMP	The survival rates are 97.4 ± 2.4%, 83.1 ± 5.2%, 63.8 ± 4.2%, 33.8 ± 3.7%, and 27.4 ± 3.1% after 0, 30, 60, 90, and 120 s insonation, respectively. Apoptotic rate was > 35% in SDT group vs < 5% in control group
					Intensity: 1 W/cm^2^		
					Timing: varied from 0 to 120 s (interval of 30 s)		
Sheehan et al. [30]	*In vitro*	2020	C6 glioma cells and U87 human GB cells	To study the effects SDT using 5-Aminolevulinic acid hydrochloride (5-ALA) and high frequency focused ultrasound (FUS) on 2 GB cell lines	Frequency: 1 MHz	SDT resulted in appreciable GB cell death as compared to 5-ALA or FUS alone	reductions in cell viability for 5-ALA, FUS, and SDT groups of 5%, 16%, and 47%, respectively compared to control
					Intensity: 10 W/cm^2^		
					Timing: 3 min		
Shono et al. [31]	*In vitro*	2021	Mouse glioma stem cells	To assess the anti-tumor effects of SDT with a Cox2 inhibitor in mouse glioma stem cells	Frequency: 1 MHz	The combination therapy with SDT and celecoxib resulted in enhanced anti-tumor efficacy among GSCs and a mouse GSC-bearing glioma model	Tumor volume reduction at 14 days after SDT was > 70% vs < 40% in control group. Kaplan–Meier survival rate and survival period prolonged by the combination therapy with SDT and celecoxib
					Intensity: 2 W/cm^2^		
					Timing: 2 min		
Shen et al. [32]	*In vitro*	2021	U87MG human GB cells	To investigate the antitumor effect of SDT combined DVDMS on human GB	Frequency: 1 Hz	The DVDMS-mediated SDT could induce great cytotoxicity in GB cells via the production of ROS	The apoptosis and necrosis ratio of SDT group: 29.17 ± 3.45%. Survival cell rate was < 40% in SDT group vs > 95% in control group
					Intensity: 0.32W/cm^2^		
					Timing: 3-min

**Table 2:** Summary of ‘in vivo’ studies included in the review

References	Type of study	Publication year	Type of cells	Animal model	Aim	Parameters	Results	Outcome
Nonaka et al. [33]	*In vivo*	2009	C6 glioma cells	Wistar rats	To determine the optimal focused ultrasound acoustic conditions using the photosensitizer Rose Bengal (disodium tetraiodo tetrachloro fluorescein) for the ablation of experimental intracranial glioma in rats	Frequency: 1 MHz	The combination of SDT and Rose Bengal showed a selective antitumor effect against cerebral glioma while sparing normal brain tissues	The areas of lesions in animals with and without Rose Bengal at an intensity of 110 W/cm^2^ for 5 min were 1.84 ± 0.18 and 4.47 ± 0.70 mm^2^, respectively (p < 0.05)
						Intensity: 25 W/cm^2^		
						Timing: 5 min		
Ohmura et al. [34]	*In vivo*	2011	C6 glioma cells	Wistar rats	To investigate the antitumour effect of SDT in experimental rat glioma by using focused ultrasound in combination with 5-ALA used as a sonosensitizer	Frequency: 1.04 MHz	SDT with 5-ALA and focused ultrasound achieved selective antitumour effect against deep-seated experimental glioma	The largest tumour areas found were 29.94 ± 10.39, 30.81 ± 9.65, 32.98 ± 7.21 and 18.32 ± 5.69 mm^2^ in the rats undergoing sham operation, ultrasound irradiation alone, 5-ALA alone and SDT, respectively
						Intensity:10,15, 20 or 25 W/cm^2^		
						Timing: 5 min		
Jeong et al. [35]	*In vivo*	2012	C6 glioma cells	Sprague Dawley Rats	To evaluate the sonodynamically induced selective antitumor effects of 5-aminolevulinic acid (5-ALA) on a C6 glioma that was implanted in a rat brain	Frequency: 1.0 MHz	SDT with 5-ALA and focused ultrasound irradiation was effective for the treatment of intracranial gliomas in rats, by a selective tumor destruction and tumor growth inhibition through nonthermal effects of weak ultrasound irradiation in a fractionated manner	a 67% or 53% reduction in tumor volume occurred in the groups receiving 5-ALA-SDT ultrasound irradiation either 6 h or 3 h after the 5-ALA administration, respectively
						Intensity: 2.65 W/cm^2^		
						Timing: 20 min		
Tserkoorvsk et al. [36]	*In vivo*	2012	C6 glioma cells	Rat	To investigate the effect of SDT using Photolon as a sonosensitizer for the ablation of glioma C6 tumor model in rats	Frequency: 1 MHz	The combination between ultrasounds and sonosensitizer agent leads to strong damage of tumor tissue because of developing induced chemical reactions and cavitation effect implementation in the tumor cell	Photolon + ultrasound 0.7 W/cm^2^ + Photoirradiation 100 J/cm^2^ caused an area of necrosis of 100%
						Intensity: 0.7 W/cm^2^		
						Timing: 10 min		
Song et al. [37]	*In vitro *and* in vivo*	2014	C6 glioma cells	Wistar rats	To investigate the effect of hematoporphyrin monomethyl ether (HMME)-mediated SDT on C6 gliomas implanted in rat brains	Frequency: 1 MHz	SDT treatment could inhibit the expansion of intracranial gliomas related to mechanical injury and the induction of apoptosis, mediated by the increased levels of Cyto-C, microvessels distruction, inhibited angiogenesis and increased expression of HIF1	Tumor volume: 50 mm^3^ (pre-op); 15mm^3^ vs 100 mm^3^ (1-week post-op, SDT group vs control group, respectively); 55 mm^3^ vs 140 mm^3^ (2 weeks post-op, SDT group vs control group, respectively)
						Intensity: 0.5 W/cm^2^		Survival time: > 50 days (SDT group) vs < 30 days (control group)
						Timing: 120 s		
Ju et al. [38]	*In vitro *and* in vivo*	2016	SNB19 and U87MG glioma cells	BALB/c nude mice	To investigate whether hyperthermotherapy (HT) could improve the efficacy of SDT in Xenograft tumor in nude mice model	Frequency: 1 MHz	The combination of SDT, HT and sonosensitizer produced a reduction of cell viability, increased apoptosis through increased ROS production, loss in MMP, higher protein levels of Bax and cleaved caspase-3, 8, and 9 and significantly lower protein level of bcl-2	the tumor volume in HT plus SDT group is much smaller compared with SDT group (P < 0.05)
						Intensity: 2.0 W/cm^2^		
						Timing: 10 min		
Suehiro et al. [39]	*In vitro *and* in vivo*	2018	U87 glioma cells and U251 glioma stemlike cells	Immunodeficient mice	To investigate the antitumor activity of SDT combined with a sonosensitizer, 5-aminolevulinic acid (5-ALA), on malignant gliomas	Frequency: 3-MHz	5-ALA-SDT inhibited cell growth, changed cell morphology, and induced apoptosis; *in vivo* 5-ALA-SDT with HIFU prolonged survival of the tumor-bearing mice	Authors demonstrated that the mice treated with 5-ALA-SDT by using HIFU survived much longer than either the untreated mice (control) or the mice treated with HIFU alone
						Intensity: 2 W/cm^2^		
						Timing: 3 min		
Pi et al. [40]	*In vitro *and* in vivo*	2019	U87 MG-Red-FLuc human GB cells	Male Balb/c nude mice	To study the anti-tumor effect of sinoporphyrin sodium (DVDMS)-mediated SDT on nude mice bearing intracranial U87 MG-Red-FLuc human GB	Frequency: 0.996 MHz	DVDMS-mediated STD leads to an anti-proliferation effect and cell apoptosis induction	the bioluminescence photon emission value of SDT w/DVDMS + FUSiBBBo group (1.59 ± 0.68 × 10^7^ photons/s) was significantly lower than the control group (115.1 ± 97.6 × 10^7^ photons/s) on the 23rd day after tumor implantation
						Intensity: 1.7 W		
						Timing: 1 min		
Sun et al. [41]	*In vitro *and* in vivo*	2019	C6 glioma cells	Balb/c mice	To evaluate the efficacy of nanosensitizer-DVDMS (sinoporphyrin sodium) loaded iRGD modified liposomes (iRGD-Lipo-DVDMS) as a potential drug delivery system, combined with low-intensity focused ultrasound (FUS) for glioma SDT	Frequency: 1 MHz	The combination of DVDMS-SDT produced an anti-tumoral effect through increased cell apoptosis	the median survival time of the glioma bearing mice treated with iRGD-Lipo-DVDMS-SDT (40 days) was obviously longer than that of those treated with only saline (15 days), free DVDMS (19 days), or LipoDVDMS-SDT (24 days)
						Intensity: 0.4, 0.6 and 0.8 W/cm^2^		
						Timing:1 min		
Yoshida et al. [42]	*In vitro *and* in vivo*	2019	F98 glioma cells	Female Fisher rats	To investigate the efficacy of 220-kHz TcMRgFUS combined with 5-aminolevulinic acid (5-ALA) on malignant glioma *in vitro* and *in vivo*	Frequency: 220 kHz	FUS/5-ALA combination reduced cell viability by inducing apoptosis and suppressed tumor proliferation and invasion as well as angiogenesis *in vivo*, while causing minimal damage to normal brain tissue	Viability was decreased by the combination of FUS and 5-ALA at a total energy 3000 J relative to irradiated cells in the other groups (p < 0.01), reflecting a total energy dependence for the cytotoxic effects of SDT
						Intensity: 10-20W		
						Timing: 120–240 s		
An et al. [43]	*In vitro *and* in vivo*	2020	U-118 MG and U-87 MG human glioma cells	BALB/c nude mice	To explore the antitumor effects of sinoporphyrin sodium (DVDMS)-mediated photodynamic therapy (PDT) and SDT in glioma	Frequency:1.0 MHz	DVDMS-mediated PDT and SDT inhibit the proliferation of glioma cells and induce apoptosis, inhibit tumor development in U-118 MG xenograft models, suppressed PCNA and Bcl-xL levels	the fluorescence intensity of DVDMS was lower in the PDT and SDT groups compared with the DVDMS group, while tumor cell proliferation and weight were lower in the PDT and SDT groups than in the control group
						Intensity: 500 mW/cm^2^		
						Timing:1 min		
Prada et al. [44]	*In vivo*	2020	C6 glioma cells	Sprague–Dawley rats	To demonstrate the efficacy of SDT with fluorescein (FL) and low-intensity focused ultrasound in inhibiting the growth of ectopic gliomas implanted in the rat's subcutaneous tissue	Frequency: 350 kHz	SDT significantly inhibited outgrowth of ectopic C6 gliomas across FUS exposure conditions	SDT significantly restricted tumor outgrowth across all three FUS exposure conditions compared to fuorescein alone (p = 0.001, p < 0.0001, and p < 0.0001for FUS1x, 1.5 × , and 2 × + Fluo, respectively)
						Intensity: 2–3 W/cm^2^, or 3–4.5 W/cm^2^, or 4–6 W/cm^2^		
						Timing: 10 ms		
Raspagliesi et al. [45]	*In vivo*	2021	C6 glioma cells	Pigs	To demonstrate the safety of low-intensity ultrasonic irradiation of healthy brain tissue after the somministration of a sonosensitizer	Frequency: Low frequency	SDT and the use of Na-FL or 5-ALA as sonosesitizer are safe towards healthy brain tissue	Upon histopathological examination, the targeted regions did not exhibit any notable pathological abnormalities. Both neurons and glial cells were found to be intact, with no significant cellular alterations observed
						Intensity: 2–3 W/cm^2^		
						Timing: 20 min		

Using “Sonodynamic Therapy“ AND “Glioblastoma” and “Focused Ultrasound” AND “Glioblastoma” as MeSH terms, another research on ClinicalTrial.Gov was performed, identifying a total of 11 trials. After exclusion criteria were applied, only four trials were included in the review.

The characteristics of included trials are the following ones: title of the trial, identifier, status, interventions characteristics, aim of the study and locations of the trial (Table 3).

**Table 3:** Ongoing clinical trials on SDT in Glioblastoma

Title	Identifier	Status	Interventions	Aim	Locations
Sonodyamic therapy with exablate system in glioblastoma patients (sonic ALA)	NCT04845919	not yet recruiting	5-Aminolevulinic acid [5-ALA]	To evaluate the safety and feasibility of SDT with 5-aminolevulinic acid in patients with newly diagnosed cerebral GB using the ExAblate Model 4000 Type-2 “Neuro-System”	Fondazione I.R.C.C.S. Istituto Neurologico Carlo Besta, Milano
Study of Sonodynamic Therapy Using SONALA-001 and Exablate 4000 Type 2 in Recurrent GB	NCT05370508	Recruiting	SONALA-001 (ALA) and exablate device	To evaluate the safety, dose-limiting toxicities, recommended Phase 2 schedule, and preliminary efficacy of SDT using SONALA-001 and Exablate Type-2 device in subjects with recurrent or progressive GB	Ivy Brain Tumor Center, Arizona and MD Anderson Cancer Center,Houston
Study to Evaluate 5-ALA Combined with CV01 Delivery of Ultrasound in Recurrent High-Grade Glioma (HGG)	NCT05362409	Recruiting	5-Aminolevulinic acid [5-ALA]	To evaluate the Safety and Tolerability of 5-aminolevulinic Acid (5-ALA) Combined with CV01 Delivery of Ultrasound for SDT in Patients With recurrent HGG	Northwell. United States, New York
Study of Sonodynamic Therapy in participants with recurrent high-grade glioma (HGG)	NCT04559685	Recruiting	SONALA-001 (ALA)	To evaluate the ascending energy doses of SDT utilizing the Magnetic Resonance focused Ultrasound (MRgFUS) combined with intravenous 5-ALA and efficacy in patients with recurrent HGG	St. Joseph's Hospital and Medical Center

### Study characteristics

#### In vitro' and in vivo' studies

Tumor cell lines used in the listed studies were both murines and humans: rat C6 glioma cells were the most used ones, followed by human U87 GB cells and other cell lines such as human glioma cells U373, U105MG and U251MG.

‘In vivo’ studies used as animal models mainly murines, both mice and rats; only one study employed a porcine model.

Both ‘in vitro’ and ‘in vivo’ studies used different types of sonosensitizers, such as 5-Aminolevulinic acid hydrochloride (5-ALA), sinoporphyrin sodium (DVDMS), hematoporphyrin monomethyl ether (HMME), temozolomide (TMZ), photofrin, fluorescein (FL) and disodium tetraiodo tetrachloro fluorescein (Rose Bengal), as a way of increasing the tumoral cells’ vulnerability to focused ultrasounds’ exposition.

‘In vitro’ studies focused their attention on the effect that ultrasound, with or without the use of a sonosensitizer, provoked on tumoral cell lines, in term of apoptotic rate and intracellular level of reactive oxygen species (ROS) post-exposition; moreover, the minimal intensity of ultrasound in order to produce an apoptotic effect on tumoral cell was also investigated [23]. Some studies also tried to quantify the anti-tumoral effect of ultrasound with or without the use of a sonosensitizer [26].

Moreover, Gonzales et. al. proved the increased efficacy of STD in combination with bleomycin, in its inhibition of tumoral growth.

‘In vivo’ studies used an animal model to verify the feasibility of this technique: more than investigating just the anti-tumoral effect of STD, these studies prove the safety of SDT towards healthy brain tissue [33, 45].

Other information obtained from ‘in vivo’ studies regard the efficacy of focused ultrasound used in combination of a sonosensitizer in inducing tumor growth inhibition and the underlying physiopathological mechanism, described thanks to post-autoptic histology. STD therapy was able to induce an increased apoptotic rate, through an increased ROS production, reduced production anti-apoptotic/pro-angiogenic factors and microvessel destruction [37].

Many of the ‘in vivo’ studies were coupled to in vitro experiments where the same method was tested, reporting analogies and differences in both results; some studies, instead, were performed directly ‘in vivo’, on animal models. The studies reviewed used similar sonication parameters regarding intensity of sonication performed (range from 0.2 to 25 W/cm^2^). The frequency used in the studies ranged from 0.5 to 3 MHz. The maximum value of the duration was 20 min. All the studies included, especially ‘in vivo’ studies, have demonstrated that SDT are effective in reducing tumor volume, because of its high selectivity, low toxicity, and deep penetration, focusing on both the ability to reduce tumor growth and placing emphasis on the survival of tumor cells after the treatment (Tables 1, 2).

#### Clinical trials

We identified four clinical trials about SDT in GB treatment. Between them, it is listed a non-randomized, single-arm study whose purpose is to evaluate the safety and feasibility of SDT with 5-aminolevulinic acid in patients with newly diagnosed cerebral GBs using the ExAblate Model 4000 Type-2 Neuro System.

Another clinical trial, non-randomized, tried to assess the safety, dose limiting toxicities, and preliminary efficacy of SDT using SONALA-001 and Exablate Type-2 device in subjects with recurrent or progressive GB.

Additionally, a phase 1 multi-center trial started to understand the safety and tolerability of 5-aminolevulinic acid (5-ALA) combined with CV01 delivery of ultrasound for SDT in patients with recurrent high-grade glioma.

Finally, we report a phase 0 single-center open label study whose intention is to appraise the ascending energy doses of SDT utilizing the MRgFUS combined with intravenous 5-ALA and its efficacy in patients with recurrent HGG.

Studies characteristics and aim were summarized in Table 3.

## Discussion

Conventional therapeutic options in the treatment of solid brain tumors, and GBs in particular, are based on the assumption that these cancerous lesions have relatively homogeneous spatiotemporal characteristics. However, recent advances in the molecular, genetic, and epigenetic fields have shown how this does not reflect the facts at all, underscoring the inherent limitations of radiation and chemotherapy [46–48]. Moreover, the notion that GBs do not represent a focal pathological entity, but rather a pathology spread throughout the entire brain, makes clear the inherent limitations of surgery, although it still represents the therapeutic mainstay toward these tumors [49, 50].

Hence the need to develop new therapeutic strategies capable of eradicating the underlying pathology, possibly in the least invasive way, and increasing OS and PFS while safeguarding patients' quality of life and neurological status [51, 52].

In this context, the use of ultrasound for therapeutic purposes (the so-called Theranostics) appears to offer interesting potential and promising results and uses [53].

As a matter of fact, focused ultrasound can be employed either to destroy cancerous cells by heating or as an adjuvant therapy, in combination with chemotherapy or radiation therapy. The main points of values of FUS are the non-invasiveness, incision-free, controllability via real-time MR guidance and the capacity to activate the immune system [54]. The first non-invasive thermal ablation of a brain tumor in human was realized by Coluccia et al. [55] in their ongoing clinical phase I study in 2014, when they firstly employed Magnetic resonance-guided focused ultrasound surgery (MRgFUS) for safe thermal ablation of a centrally located recurrent GB. This is possible thanks to recent advances in magnetic resonance imaging, which allow safe and precise thermal ablation of neoplastic tissue. Moreover, the opportunity to create an MRI-derived temperature mapping of the targeted tissue allow a non-invasive monitoring of the ablating procedure. In more recent years, knowledge about the different mechanisms of action of ultrasound at various intensities and frequencies, used alone or in combination with other substances, has been expanded, exploring new potentials, and developing new therapeutic strategies, including precisely SDT [56–61].

### Sonodynamic therapy

SDT has been developed as a promising tool in brain tumor treatment. SDT takes its cue from photodynamic therapy (PDT), in which a light-activated photosensitizer can cause the generation of ROS, which in turn would mediate a cytotoxic effect on neoplastic cells. However, the main limitation of PDT is the range of action, which is limited to superficial lesions due to the poor penetration of laser light into brain tissue [42, 62]. This obstacle is overcome using low-intensity ultrasound, which has a greater penetrative capacity [63].

As just mentioned, SDT involves the application of focused ultrasound with a substance that sensitizes cells to the destroying effects of sound, called sonosensitizer. It includes both ultrasonication, via non-invasive low-intensity ultrasound penetrating soft tissues and focus on a specific site, and sonosensitizers, which embrace non-toxic chemical agents such as 5-ALA, ATX-70, Hypocrellin, Rose Bengal and many others [64–66]; some of these compounds are commonly used in glioma surgery to intraoperatively visualize the tumor and can be employed to induce cytotoxic effects to neoplastic cells when subjected to a specific acoustic field [31, 39, 40]. The advantage of this technique is to minimize adverse events and maximizing on-target responses. Furthermore, the use of chemical agents that are non-toxic in the absence of a specific stimulus distinguishes the definition of SDT from the broader meaning of FUS employed to enhance the effects of an already toxic compound [67, 68].

Sheehan et colleagues [69] employed the SDT on two cellular lines, rat C6 and human U87 GB cells, and found that two innocuous agents, which are FUS and 5-ALA, can lead to cell death by the transformation of 5-ALA to PPIX in malignant glioma cells, where it generates reactive oxygen species responsible of cellular apoptosis. ‘In vivo’ studies have proved that focused ultrasound in combination with the systemic administration of 5-ALA is effective in treating intracranial gliomas in rats, not demonstrated by the complete tumor resection but by the reduction in tumor size from the initial tumor volume [34, 35, 37, 39]. In this regard, Nonaka et al*.* [33] pinpointed the optimal focused ultrasound acoustic energy and duration for the ablation of brain tumor in rats, without damaging normal brain tissue; in their experience, this selective anti-tumor effect was produced by weaker focused ultrasound intensity at 25 W/cm^2^ at 1 MHz for 5 min.

In 2019, Abdolhosseinzadeh et al. [70] investigated the effects of focused acoustic waves in a focal area through some accurate simulations. Results obtained in 2D, and 3D models showed that ultrasound waves could be used in the form of pulse waves with different time periods to provoke a focused thermal lesion on neoplastic tissue [71, 72]. Nevertheless, ‘in vivo’ it is necessary to overcome the blood brain barrier (BBB), which represents a real obstacle to sonosensitizers. To this aim, low-intensity focused ultrasound can be combined to microbubbles, which proved to increase the permeability of the BBB, allowing the treatment of intracranial GB in mice. These results can suggest the use of SDT with sonosensitizers in human GB [73–75].

### Sonosensitizers

As previously mentioned, the sonosensitizers used in SDT are harmless molecules that when subjected to an acoustic field mediate a cytotoxic effect. Many of these molecules are the same as those used in photodynamic therapy and are agents based on porphyrin or related molecules (protoporphyrin IX, hematoporphyrin, etc.). In fact, there is evidence that such molecules, when exposed to the action of ultrasound, result in the production of reactive oxygen species (ROS). In their *‘*in vitro*’* study, Shen et al*.* [32] employed as a sonosensitizer the sinoporphyrin sodium, purified from photofrin II, which showed great antitumor effect on human GB cell lines; particularly, this sonosensitizer can easily enter in cancer cells and accumulate into the mitochondria, where it gives raise to cytotoxity through the production of ROS.

However, although these agents are preferentially picked up by the tumor, they exhibit marked hydrophobicity, and their distribution appears to be ubiquitous [76]. Despite this apparently drawback, it has been postulated by Raspagliesi and colleagues that three contemporary events must occur to determine a cytotoxic effect: the administration of ultrasound, the administration of a sonosensitizer, and the presence of a lesion where the latter can reach a significant concentration. This concept led to the non-invasive effect on SDT on normal brain tissue, since, even if the sonosensitizer has been collected in healthy tissue, it would be inconsequential [45].

Thus, it seems clear that the choice of sonosensitizer is also crucial. Ideally, the perfect sonosensitizer should exhibit high affinity for tumor cells and slow clearance from the neoplasm, while sparing healthy brain parenchyma [77–79].

### Mechanism of action of sonodynamic therapy

**Fig. 2:**
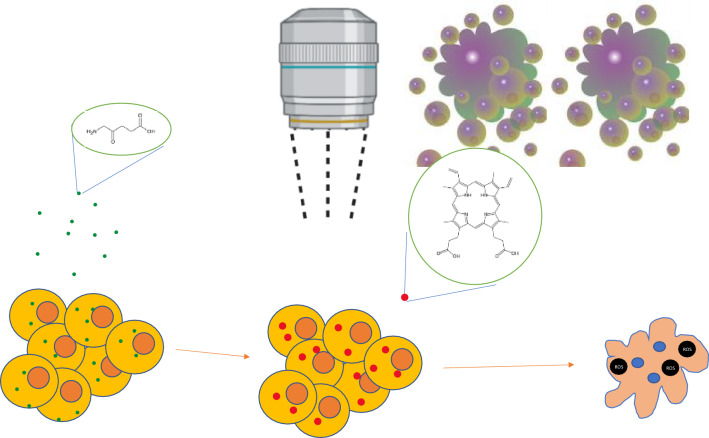
Sonodynamic therapy could be effective in glioma cells-death though transformation of 5-ALA (green dots) to PPIX (red dots). The process results in the production of reactive oxygen species (ROS), leading to cells death

**Fig. 3:**
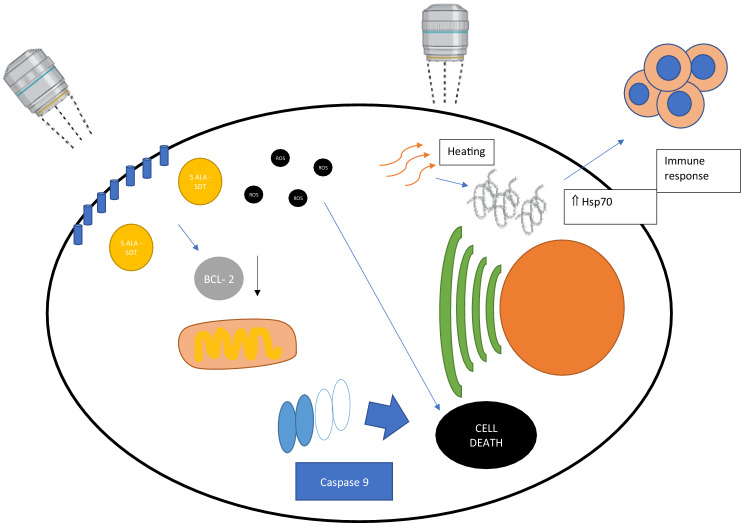
Representation of 3 mechanisms of sonodynamic therapy: **A** 5 ALA SDT could reduce level of Bcl2, thus activating apoptosis via caspase 9 pathway. **B** 5 ALA SDT increase levels of ROS, thus inducing cell death. **C** heating itself could increase level of Heat shock protein 70, thus inducing immune response through structural changing in cell membrane

The effects of thermal ablation on tumoral cells are still not completely clear. Is has been demonstrated that hyperthermia (HT) can enhance the 5-ALA-SDT induced cell apoptosis partly by activating caspases and by modulating Bcl-2 family members. Moreover, HT is responsible of increasing ROS production and reducing metalloproteases (MMPs) induced by 5-ALA-SDT in human glioma cells [38, 80] (Figs. 2, 3).

HT can also regulate some molecular aspects of the immune response, such as Fas gene and its ligand FasL, and act as an immunomodulator in cancer therapy [81, 82]. In more details, it seems that the increasing in local temperature may act as a natural trigger or danger signal to the immune system. Hyperthermia can therefore enhance the expression of FAS-ligand mRNA, which has a role in functional maturation of dendritic cells together with secretion of proinflammatory cytokines, which in turn activate T lymphocytes and induce a polarization toward a Th1 phenotype. Moreover, high temperature may promote the action of a particular set of protein, the HSPs, which may act in protecting cells from dangerous stress by regulating cell homeostasis [83] and may also affect the stability of cellular membranes by inducing structural changing that intervene in signaling events and cell migration in immune response [84, 85].

The link between ultrasound exposure, presence of sonosensitizer and generation of ROS appear clear, and there is a consensus regarding their involvement in mediating the cytotoxic effect on cancer cells [86–88]. Nonetheless, other mechanisms have been proposed to elucidate the SDT-mediated cytotoxic effect. These include sonoluminescence, namely the emission of light from cavitation bubbles, which would appear to play a role both in the activation of certain sonosensitizers and in mediating antitumor effects [89, 90], and sonomechanical mechanisms that would mediate damage by inducing changes at the level of cell membranes, such as a reduction in membrane fluidity and an increase in lipid peroxidation [91, 92]. Noteworthy are the various cytotoxic actions and implicated mechanisms that characterize the different sonosensitizers, although further studies on this are needed [93, 94].

Interestingly, SDT can also be used in combination with other agents commonly used to treat GB to enhance their action, such as the temozolomide (TMZ). Resistance of high-grade glioma cells to TMZ is related to high level expression of NHE-1 protein, which enhance cells invasion to normal brain tissue. In their article, Chen et al. [27] demonstrated that SDT can suppress NHE-1 expression, thus allowing the cytotoxic effect of TMZ in vitro.

Another target to take advantage of to enhance the anti-tumor efficacy of TMZ is the p-glycoprotein, referred to as multidrug resistance receptor (MDR1), a transmembrane protein which act as an efflux pump and confer multidrug resistance in brain tumor. Consequently, high expression of MDR1 is present in resistant GB and the downregulation of MDR1 via Akt/NF-kB pathway can improve the antitumor effect of temozolomide in GB cells. Shono et al*.* [31] demonstrated that the elevation of cellular PpIX using celecoxib is related to a down regulation of Akt/NF-kB/MDR1 pathway, thus enhancing the anti-tumor efficacy of SDT. Some authors advocated that the SDT mediated by hematoporphyrin monomethyl ether (HMME) can induce apoptosis on C6 glioma cells *in vitro* and suggest that the mitochondrial signal pathway may play a pivotal role, because of the observed production of ROS, loss of MMP and Bcl-2 and protein expression in caspace-9, caspase-3 and Bax [25, 29].

### Ultrasound parameters affecting SDT results

Although the exact mechanism of action of SDT is not yet fully understood, it is assumed that the biological effects of this technique are strongly correlated with the phenomenon of acoustic cavitation (stable vs. inertial cavitation) derived from the interaction between ultrasound and the propagation medium, ultimately resulting in apoptosis of the affected cells. In addition to the mechanical effect of ultrasound, the action of SDT is also based on the sonochemical effect related to the formation of various species of free radicals and the different decomposition kinetics of sonosensitizers [63, 95, 96]. These various mechanisms of action in turn are closely related to the ultrasound parameters used and to other factors associated to the experimental setting (see Table 4).

**Table 4:** ‘In vitro’ factors and variables potentially influencing SDT outcomes

Factors	Variables
Ultrasound Parameters	• Frequency
	• Intensity
	• Irradiation time and duty cycle
Spatial Configuration	• Cell-to-transducer distance
	• Ultrasound beam/culture vessel ratio
Coupling Media	• Volume
	• Composition
	• Viscosity
	• T°
	• Acoustic Propagation
Culture Vessels	• Geometry
	• Absorbers
	• Material Type
Culture Medium	• Composition
	• Volume
	• Acoustic Properties
Irradiation sample type	• Adhering cells
	• Suspension cells
	• Cell motion

For instance, it has been demonstrated that most sonosensitizers respond to US frequency ranging from 0.2 to 3 MHz [42] and that a decrease in frequency is correlated to an increase in ultrasound toxicity [25]. However recent works have pointed out apoptotic cell ratio was primarily affected by sonosensitizer concentration and then by other variables such as US frequency, irradiation time and intensity [97, 98]. US intensity usually ranges from 0.5 to 10W/cm^2^ and can be applied in a continuous or pulsatile mode [96]. Regarding this parameter, many studies have noted an intensity-dependent reduction in cell viability of various cancer types [99, 100]. Nejad et al. [101] have shown in a model of human oral squamous cell line HSC-2 how irradiating cells with 3.5 MHz US at 20, 32, 55, and 73 W/cm^2^ was associated to a cell survival rate of 97, 81, 62 and 40%, respectively.

Irradiation time and duty cycle also influenced SDT results, with greater citotoxity at greater irradiation time and duty cycle [102]. Besides US parameters, many other factors may influence SDT response ‘in vitro’ studies. Irradiation uniformity and intensity distribution, for example, are related to the distance between cells and US apparatus, as well as the characteristics of the coupling media and culture medium also seem to profoundly impact the outcomes of SDT. Other two crucial factors that affect the results of SDT are the type of irradiated sample and the sonosensitizer concentration, the latter closely related to the apoptotic effect, as shown by Zhang et al. [103–105].

### Ongoing clinical trials

Evidence regarding the potential application of SDT in high-grade gliomas are mostly taken from pre-clinical ‘in vitro’ and ‘in vivo’ studies. Currently, there are four ongoing trials concerning the role of SDT in high grade gliomas registered on “Clinicaltrial.gov”, of which three are recruiting.

The aim of the first trial is to evaluate the safety and tolerability of 5-ALA combined with CV01 delivery of ultrasound in patients affected by recurrent high-grade gliomas. (ClinicalTrials.gov Identifier: NCT05362409). This ongoing phase 1 trial is recruiting 33 patients, to which 5-ALA will be administered as sonosensitizer prior to CVo1-delivered ultrasound, which will deliver non-ablative, low-intensity ultrasound; 5-ALA will be then re-administered every 4 weeks prior to CV01. The primary outcome is to evaluate the incidence of adverse events and to determine the Maximum Tolerable Duration in the first 12 months. Secondary outcome is represented by the assessment of Overall Response Rate, Duration of Response, OS and PFS in the first 12 months.

The second recruiting trial (ClinicalTrials.gov Identifier: NCT05370508) aims to evaluate the safety and preliminary efficacy of SDT by using SONALA-001 as sonosensitizer and Exablate 4000 Type-2 MR-guided focused ultrasound as device in people affected by recurrent or progressive GB. The primary outcomes are represented by the evaluation of the safety of SDT in the first 12 months, the maximum tolerable duration in the first 29 days, the determination of recommended phase 2 schedule and the assessment of progression free survival in the first 6 months.

The third prospective, non-randomized, single-arm, not yet recruiting study (ClinicalTrials.gov Identifier: NCT04845919) aims to evaluate the safety and feasibility of SDT by using 5-ALA and Exablate 4000 Type-2 MRgFUS in patients newly diagnosed with GB. Patients screened will undergo SDT treatment, will perform a strict neuro-radiological follow-up after the procedure (minimum 2 MRI) and will undergo tumor resection 14–21 days after SDT. The primary outcome is represented by the early identification of hemorrhage, oedema, or other damages in the first 10 days; secondary outcomes are represented by the evaluation of the rate of neurological deficits and the radiological response to treatment in the first 10 days after the procedure.

The last nRCT (identifier: NCT 04559685) is a Phase 0 single center, first in human, open-label study of ascending energy doses of SDT utilizing the MRgFUS combined with intravenous ALA to assess safety and efficacy in up to 30 participants with recurrent HGG. The primary outcomes are to assess the biological changes associated with the SDT, analyzing the percentage of Cleaved Caspase-3, MIB-1 level and GammaH2Ax of the surgical specimen. Secondary outcomes include the evaluation of radiographic evidence of tumor physiological imaging changes and the assessment of performance, safety and tolerability of the MRgFUS and SDT.

## Conclusions: challenges, limits, and future directions

This review explored the current literature regarding the role of SDT in glioma treatment, and particularly in GB, considering the evidence from ‘in vitro’ and ‘in vivo’ studies, and the ongoing clinical trials on its clinical human application. We also focused on the possible mechanisms of action underlying SDT and the role of different sonosensitizers. The study of the latter seems to enshrine the marriage of SDT and nanomedicine, paving the way for future research and new possibilities.

Based on the studies that have been discussed on this paper and the current ongoing trials, SDT could be a valuable option in patients with GB, due to the opportunity to induce toxicity only in a precise localization while minimizing harm in normal areas. Indeed, thanks to the development of increasingly sophisticated and accurate software is possible to target tumor volume precisely. In addition, nanotechnology-based drug delivery systems have been developed to enhance the selective accumulation of the sonosensitizer in tumor cells. Actually, SDTseems to be more effective in treating GB than low-grade glioma (LGG). This is because GB cells are more susceptible to the effects of SDT due to their higher rate of metabolism and greater degree of angiogenesis compared to LGG cells [38, 106, 107]. However, more research is needed to confirm these findings and determine the optimal parameters for SDT in the treatment of different types of brain tumors. Furthermore, the effectiveness of SDT may also depend on other factors such as tumor size, location, genetic characteristics, and vascular pattern. For instance, brain tumors located near the skull base may be more amenable to SDT. The size of the tumor can also affect the effectiveness of the procedure. Larger tumors may be more difficult to treat with SDT, as the ultrasound waves may not be able to penetrate deep enough into the tumor to effectively kill the cancer cells.

Limitation on the clinical application rely on the fact that SDT represents a novel technique that needs to be further investigated. First, the role of sonosensitizers should be deepened: many sensitizers are employed in both PDT and SDT and residuals can accumulate in areas other than tumors, thus leading to hypersensitivity to light. Strategies to overcome this limitation are therefore needed, such as the opportunity to employ microbubbles to carry sonosensitizer or to employ new sensitizers specific for the SDT. Moreover, attention should be payed to the phenomenon of cavitation, which enables the sonochemical reactions to occur. Initiation of cavitation can be difficult, because of the high pressure required. Authors suggested some strategies to facilitate the cavitation, such as the application of standing waves rather than progressive or the dual frequency sonication [96].

Other major concerns are that US procedures require long treatment sessions, therefore confining its application to small volumes, and the lack of in-vivo studies; in this regard efforts have been made and several ongoing clinical and pilot trials aim to better define the real clinical employment of SDT in patients affected by GB [108].

Challenges are also represented by the correct application into the neurooncological field of devices currently employed for other neurological disorders; an example is given by the essential tremor which benefit from the MRgFUS performed with the ExAblate Neuro 4000. It is therefore necessary to adapt and modify some characteristic such as the frequencies employed, which are lower in the setting of a tumor compared to the treatment of essential tremor (220 KHz vs 650 KHz) [109].At present, SDT is not indicated as a first-line treatment for GB or any other type of brain cancer.

However, several scenarios are still open: SDT may have potential as an adjunctive treatment to enhance the effectiveness of standard therapies, such as chemotherapy via chemosensitization effect, or as a salvage option for patients who have failed other treatments, alone or in combination to other techniques to enhance its effect (e.g. hyperthermotherapy). In some cases, it may also be considered as a primary treatment strategy for patients who are not suitable candidates for surgery or who have recurrent tumors that are difficult to treat with other modalities.

Further studies are certainly needed to better define the role of sonodynamic therapy in these patients and, particularly, the eligibility criteria for this treatment, such as the stage of disease (i.e., primary, or recurrent GB) and the opportunity to employ SDT as a first line treatment or as a palliative strategy, as well as patient condition, such as KPS or current comorbidities. Finally, the opportunity to use this technique in brain tumors other than gliomas should be deepened: over the last years new indications have been considered as potential targets of the ultrasound therapy, such as brain metastasis (from breast cancer or melanoma), neuroblastoma, neurofibromatosis, astrocytomas and pontine gliomas, and both preclinical and clinical trials are ongoing [108].

## Data Availability

All data generated or analyzed during this study are included in this published article.
